# Laser capture microdissection for transcriptomic profiles in human skin biopsies

**DOI:** 10.1186/s12867-018-0108-5

**Published:** 2018-06-19

**Authors:** Silvia Santoro, Ignazio Diego Lopez, Raffaella Lombardi, Andrea Zauli, Ana Maria Osiceanu, Melissa Sorosina, Ferdinando Clarelli, Silvia Peroni, Daniele Cazzato, Margherita Marchi, Angelo Quattrini, Giancarlo Comi, Raffaele Adolfo Calogero, Giuseppe Lauria, Filippo Martinelli Boneschi

**Affiliations:** 1 Laboratory of Human Genetics of Neurological Disorders, Institute of Experimental Neurology (INSPE), Division of Neuroscience, IRCCS San Raffaele Scientific Institute, Milan, Italy; 2 Laboratory of Neuropathology, Institute of Experimental Neurology (INSPE), Division of Neuroscience, IRCCS San Raffaele Scientific Institute, Milan, Italy; 3 Neuroalgology Unit and Skin Biopsy, Peripheral Neuropathy and Neuropathic Pain Center, IRCCS Foundation “Carlo Besta” Neurological Institute, Milan, Italy; 4 Department of Clinical and Biomedical Sciences “Luigi Sacco”, University of Milan, Milan, Italy; 5 Department of Neurology, IRCCS Ospedale San Raffaele, Milan, Italy; 6 Molecular Biotechnology Center, Department of Biotechnology and Health Sciences, University of Turin, Turin, Italy; 7 Laboratory of Genetics of Complex Disorders and Department of Neurology, IRCCS Policlinico San Donato, Milan, Italy; 8 Department of Biomedical Sciences for Health, University of Milan, Milan, Italy

**Keywords:** Transcriptomics, Skin biopsy, RNA sequencing, Laser capture microdissection, Idiopathic neuropathy

## Abstract

**Background:**

The acquisition of reliable tissue-specific RNA sequencing data from human skin biopsy represents a major advance in research. However, the complexity of the process of isolation of specific layers from fresh-frozen human specimen by laser capture microdissection, the abundant presence of skin nucleases and RNA instability remain relevant methodological challenges. We developed and optimized a protocol to extract RNA from layers of human skin biopsies and to provide satisfactory quality and amount of mRNA sequencing data.

**Results:**

The protocol includes steps of collection, embedding, freezing, histological coloration and relative optimization to preserve RNA extracted from specific components of fresh-frozen human skin biopsy of 14 subjects. Optimization of the protocol includes a preservation step in RNALater^®^ Solution, the control of specimen temperature, the use of RNase Inhibitors and the time reduction of the staining procedure. The quality of extracted RNA was measured using the percentage of fragments longer than 200 nucleotides (DV_200_), a more suitable measurement for successful library preparation than the RNA Integrity Number (RIN). RNA was then enriched using the TruSeq^®^ RNA Access Library Prep Kit (Illumina^®^) and sequenced on HiSeq^®^ 2500 platform (Illumina^®^). Quality control on RNA sequencing data was adequate to get reliable data for downstream analysis.

**Conclusions:**

The described implemented and optimized protocol can be used for generating transcriptomics data on skin tissues, and it is potentially applicable to other tissues. It can be extended to multicenter studies, due to the introduction of an initial step of preservation of the specimen that allowed the shipment of biological samples.

**Electronic supplementary material:**

The online version of this article (10.1186/s12867-018-0108-5) contains supplementary material, which is available to authorized users.

## Background

The ability to generate tissue-specific expression profiles can significantly expand our knowledge on the pathophysiological mechanisms of different diseases, providing information on functional elements and molecular constituents of the tissues and identifying new diagnostic and therapeutic biomarkers [1]. High-throughput gene expression profiling through next generation sequencing (NGS) has several advantages over microarray technology, such as single-nucleotide resolution and the possibility to study the entire RNA content of a single cell, cell populations or tissues starting from nanograms quantities of input material [2, 3]. Moreover, by assaying millions of transcripts in RNA sequencing data, it is also possible to discover gene fusion, alternative splicing and novel isoforms expanding the complexity of gene expression field not possible with microarray technology [1, 2].

To study tissue-specific expression profile, the first step is the isolation of the biological source. The laser capture microdissection (LMD) technology allows the isolation of cells of interest morphologically identified via microscopic visualization. It can minimize the hurdle of tissue heterogeneity observed in homogenates of whole tissues [4, 5], allowing genomics, transcriptomics, and proteomics studies on accurately isolated target cells [5]. To successfully capture the sample of interest, the preparation of the specimen is fundamental [6] and needs to be methodologically implemented to obtain sufficient amount of RNA of high quality from microdissected fresh tissue for an accurate expression profiling [7–12]. Although different commercially available kits can be employed to extract RNA from microdissected sections [6], RNA preservation can be affected by the microdissection phase, the extraction procedures and the presence of nucleases in aqueous solutions or in tissues [7–10].

In the present paper, we describe an optimized protocol starting from human skin biopsy collection at the distal leg up to the final step of RNA extraction from layers of fresh-frozen sections microdissected by LMD. We implemented several optimizations to overcome the problems related to the high instability of RNA molecule, the presence of high amounts of nucleases in the skin and the many steps necessary for biopsy processing, improving the quality of extracted RNA. Moreover, we introduced an initial step of immersion of the specimen into RNALater^®^ preserving solution (Applied Biosystems, Foster City, CA, USA), making this protocol suitable also for multicenter studies in which biological specimens need to be collected by different centers and shipped for future processing in a central laboratory, allowing its use in clinical and research studies.

## Results

### Subject recruitment

Eight patients (5 males and 3 females) affected by idiopathic sensory neuropathy (mean age ± standard deviation (SD): 63.4 ± 4.2 years), and six age-matched healthy controls (4 males and 2 females; mean age ± SD: 53.8 ± 3.5 years) were recruited. Skin biopsies were collected from the 14 subjects and preserved in RNALater^®^ solution at 4 °C for 1 week.

### Cryosection, hematoxylin and eosin (H&E) staining and laser capture microdissection

We included the biopsies in Killik cryostat embedding OCT medium and, after the storage in liquid nitrogen for at least 1 week, we proceeded with the cryosection facilitated by liquid nitrogen flow. H&E staining was then performed modifying the protocol published by Yee and colleagues [9] to accelerate the staining time in order to preserve the integrity of the RNA.

**Fig. 1:**
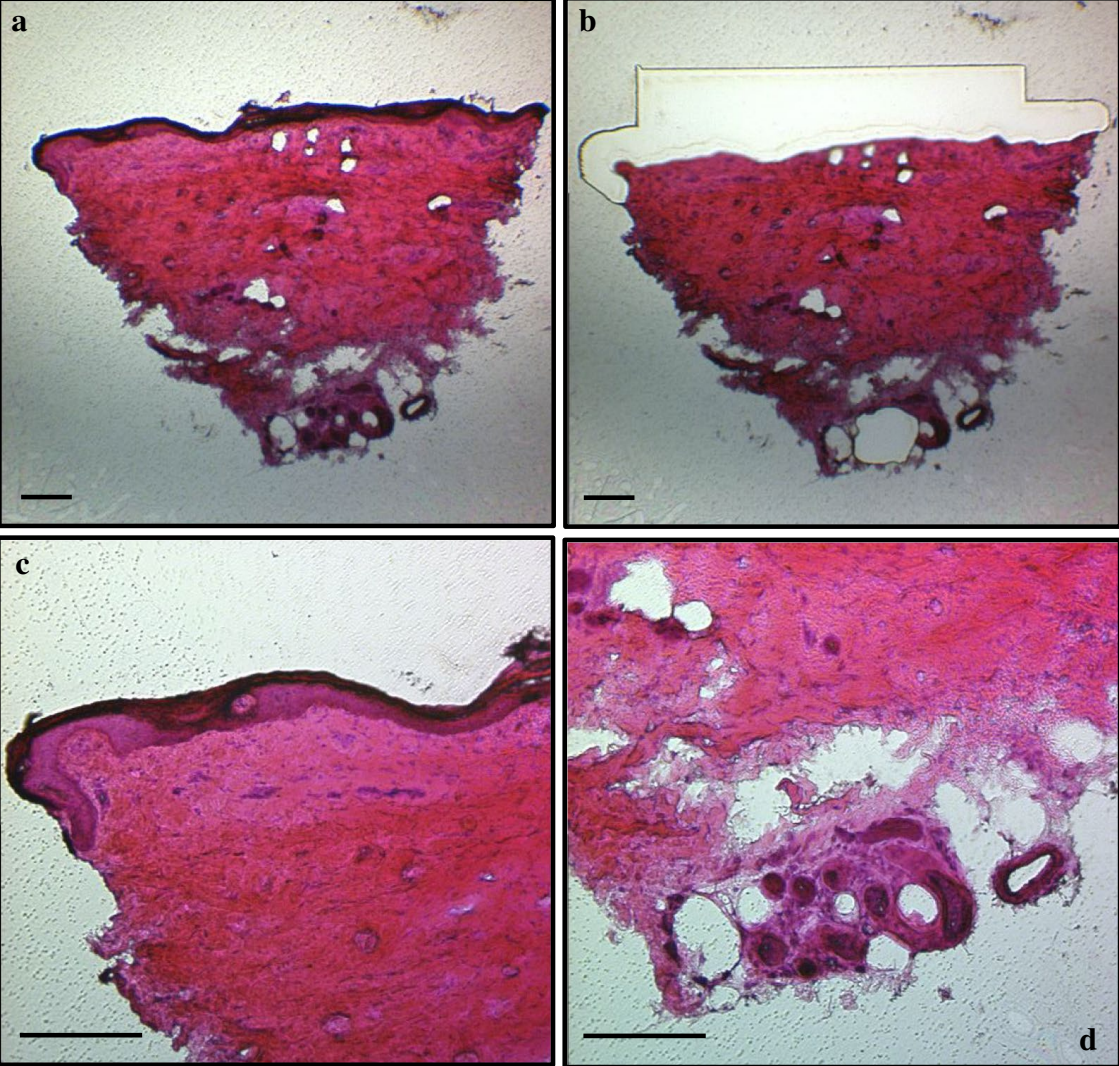
Representation of the four skin components stained with hematoxylin and eosin. **a** whole section: **b** dermis; **c** enriched layer of fibers extending for 200–300 μm from the surface layer of the skin and **d** glands. Magnification: ×4 (**a**, **b**) and ×10 (**c**, **d**). Scale bar = 200 μm

Proceeding with the LMD, two-dimensional measurements and numbers of collected areas were registered before cutting the selected area in order to calculate the volume of tissue from which RNA was extracted. The microdissected sections were the enriched layer of fibers (ELF), the glands (G), the dermis (D) and the whole section (WS). The epidermis was not separately dissected from the biopsy but it was part of the ELF and of the WS. A representative picture of the staining of the four tissue components are shown in Fig. 1. As expected, whole section was the component with the highest area and volume, while the lowest quantity was represented by glands. Measurements are reported in Table 1 as mean value ± SD of all samples for all skin components. Overall, it was possible to dissect the glands in 9 subjects (6 males, of which 4 patients and 2 healthy controls, 1 female patient and 2 female healthy controls) out of 14 because they were not present in all the skin biopsies, while the other three layers were dissected in all subjects.

**Table 1:** Measurements of microdissected skin area and extracted RNA

Tissue	ELF (n = 14)	G (n = 9)	D (n = 14)	WS (n = 14)
Number of microdissected areas (n)	12.2 ± 3.6	5.9 ± 5.9	9.9 ± 2.1	8.6 ± 1.8
Microdissected area (mm^2^)	1.1 ± 0.4	0.06 ± 0.07	1.6 ± 0.8	2.3 ± 1.1
Microdissected volume (mm^3^)	0.02 ± 0.01	0.001 ± 0.001	0.03 ± 0.02	0.05 ± 0.02
Concentration (ng/μl)	3.7 ± 2.7	2.0 ± 0.1	2.6 ± 0.7	3.1 ± 1.4
RIN	2.2 ± 0.4	1.4 ± 0.7	1.1 ± 0.3	2.6 ± 1.1
DV_200_ (%)	87.7 ± 3.4	81.0 ± 6.3	79.4 ± 9.2	87.4 ± 5.7

Summary of the number, area and volume of microdissected areas, RNA concentration, RNA Integrity Number (RIN) and percentage of fragments longer than 200 nucleotides (DV_200_) for all the samples in different skin components. All the values were reported as mean ± standard deviation (SD). *ELF* enriched layer of fibers, *G* glands, *D* dermis, *WS* whole section

### RNA quantity measurements and quality assessment

Table 1 shows the average concentration and the total amount of RNA obtained for each tissue. The highest concentration and total amount of RNA were obtained from ELF and WS, with a mean RNA concentration ± SD of 3.7 ng/µl ± 2.7 from ELF, 2.0 ng/µl ± 0.1 from G, 2.6 ng/µl ± 0.7 from D and 3.1 ng/µl ± 1.4 from WS (p: 0.004 between ELF and G). As expected, the four tissues showed a similar degradation degree that was lower than non-degraded RNA (RIN > 7). In particular, the ELF and WS reported a mean RIN ± SD of 2.2 ± 0.4 and 2.6 ± 1.1 respectively, while G and D of 1.4 ± 0.7 and 1.1 ± 0.3 respectively (mean ± SD) (Table 1). A significant difference of RIN occurred between WS vs G (p value 0.01), and WS vs D and ELF vs D (p values < 0.0001). Since the extracted RNA was partially degraded, a more suitable measurement of degradation, the DV_200_ metric defined as the percentage of RNA fragments longer than 200 nucleotides, was used to evaluate sample quality for the libraries preparation according to Illumina technical note [13]. Except for three samples of D (sample 2, 9 and 11) and one of G (sample 10), with values ranging between 61 and 67%, all samples reached a quality level of DV_200_ ≥ 70% (Additional file 1: Table S1). The mean value ± SD of all measurements are reported in Table 1, while in Additional file 1: Table S1 we presented the details of each single sample.

### Library generation and sequencing

**Fig. 2:**
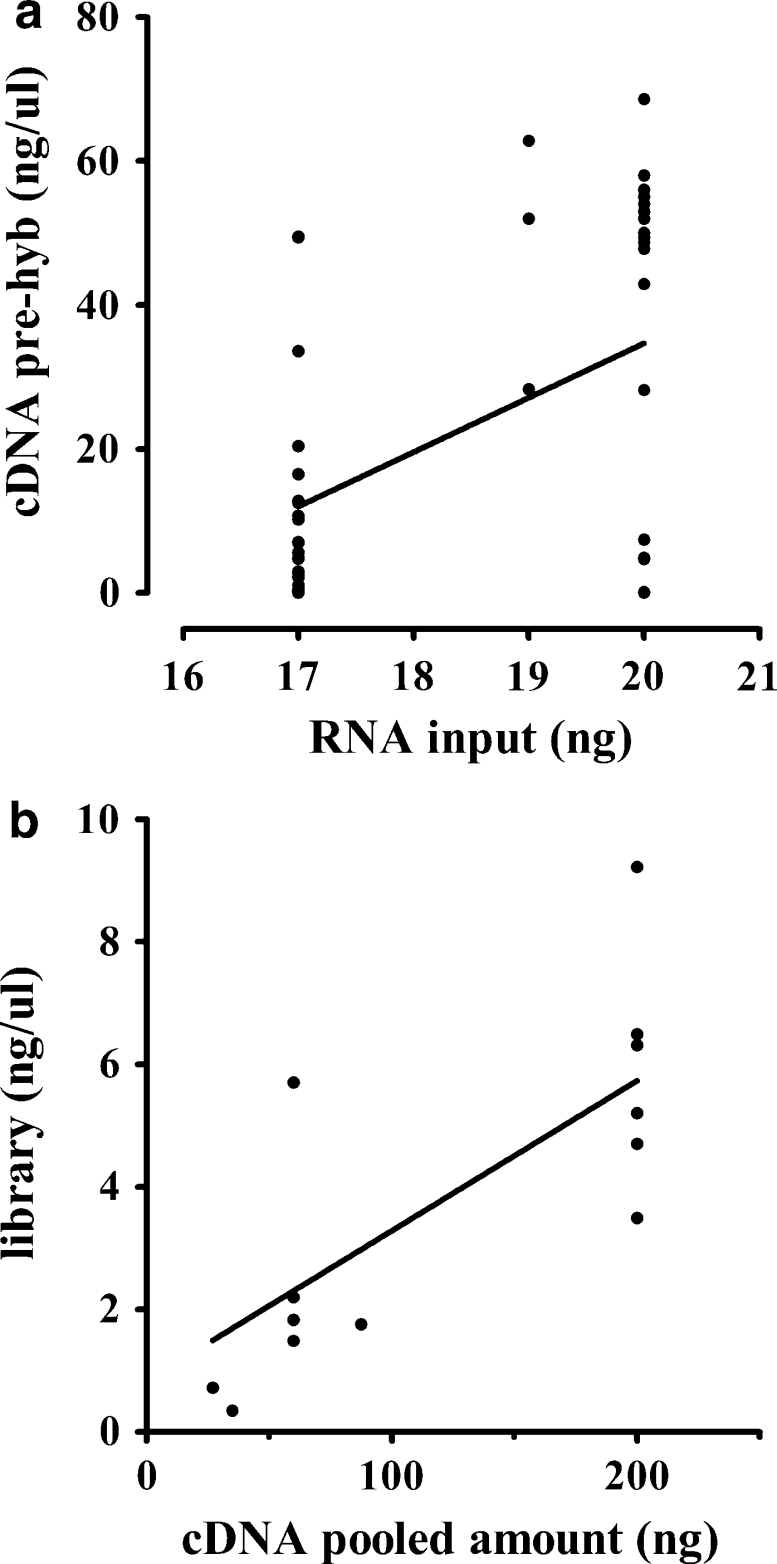
RNA/cDNA and cDNA/library correlation. Correlation between the RNA input and the yield of cDNA obtained before the hybridization step (**a**; p: 0.031, beta: 7.56 and r^2^: 0.22) and between the pooled amount used for cDNA hybridization and the yield of final libraries (**b**; p < 0.0001, beta: 0.02 and r^2^: 0.52)

As reported in Additional file 1: Table S1, library preparation was performed for 24 samples using 20 ng of extracted RNA following the manufacturer’s protocol, while for 27 samples the extracted amount was not sufficient thus we used the maximum available amount (17–19 ng). Ten samples did not reach a cDNA concentration higher than 1 ng/μl, and they were excluded from the experiment. Their exclusion seems not be related to the input amount nor to the degree of degradation (Additional file 1: Table S1). As expected, a significant positive correlation between input RNA and the yield of cDNA was confirmed by Spearman’s correlation analysis, as shown in Fig. 2a (p value: 0.031; beta: 7.56; r^2^: 0.22). Unexpectedly, for 7 of 24 samples that fulfilled the requested input of RNA it was not possible to reach 1 ng/µl of cDNA. On the contrary, for 24 out of 27 samples with a lower amount of input RNA, we obtained enough quantity of cDNA to proceed with the protocol. Hybridization occurred in multiplexed modality using 200–30 ng of cDNA from each sample depending on the reached cDNA amount. In detail, the amount for pools preparation was calculated according to the sample with the lowest concentration within each pool in order to obtain homogeneous enriched samples. Additional file 1: Table S1 reports the hybridized amount, the pooling strategy, and the final concentration of libraries. A significant positive correlation between the pooled amount and the yield of libraries was confirmed by Spearman’s correlation analysis, as shown in Fig. 2b (p value < 0.0001; beta: 0.02; r^2^: 0.52).

A number of reads between 21 and 35 million/sample was obtained in 16 samples, between 35 and 90 million reads/sample in 22 samples, and less than 20 million reads (ranging from 13.2 and 17.8 millions of reads) in 3 samples (Additional file 1: Table S1).

### Quality control of RNA sequencing data

In order to rule out potential batch effects caused by the types of samples and/or by the technique, the base sequence quality by cycle expressed with the Phred quality score (Q score) was evaluated and represented across tissue compartments (Additional file 2: Figure S1A) and flow cells (FC) (Additional file 2: Figure S1B). As shown in Additional file 2: Figures S1A, B, the Q scores of the experiment ranged from 37 to 39.5, and the trend was similar across different conditions.

To further increase the homogeneity of data, we performed a down-sampling, reducing samples with more than 45 million reads to 35 million. In Additional file 3: Figure S2 it is reported the density plot of log10-transformed reads counts. The global trend was similar across samples, and it followed a Gaussian distribution.

The Principal Component Analysis (PCA) was performed to identify potential outliers. Additional file 4: Figure S3 shows data projected onto the 3 principal components (PCs), which account for approximately 43% of the overall variance of the dataset. As shown in Additional file 4: Figure S3, a degree of separation between ELF and WS and between G and D along the second PC was observed. Additionally, sample S7d could be deemed as a potential outlier along the first PC (that explains the largest variability across samples), and the sample S11g along the third PC (that explains a lower variability across samples).

Aligning the sequenced reads to the human reference genome, we reached a mean percentage of uniquely mapped reads of 83.2% ± 1.4 (mean ± SD) across ELF, WS and D while for glands it was 82.2% ± 3.7 (mean ± SD) (Additional file 5: Figure S4A). A trend of positive correlation between the number of uniquely mapped reads and the DV_200_ was confirmed by Spearman’s correlation analysis, as shown in Additional file 5: Figure S4B (p value: 0.013; beta: 0.11; r^2^: 0.15).

**Fig. 3:**
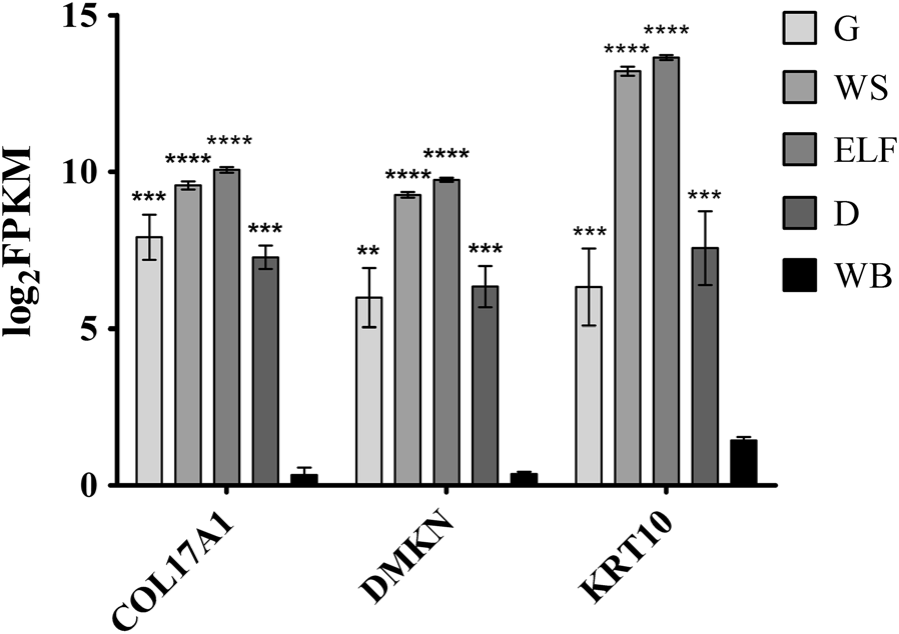
RNA expression of genes enriched in skin. Graph of log_2_ FPKM (Fragments Per Kilobase Million) values of three genes known to be enriched in skin tissue (*COL17A1*: Collagen Type XVII Alpha 1 Chain; *DMKN* Dermokine; *KRT10*: Keratin 10) evaluated in each skin compartments and in whole blood (*ELF* enriched layer of fibers, *G* glands, *D* dermis, *WS* whole section, *WB* whole blood). Bar plot shows the mean ± standard error of the mean. Statistical significance is reported for each skin compartments compared to whole blood (**p: 0.0013; ***0.0001 ≤ p ≤ 0.0007; ****p < 0.0001)

To further confirm the reliability of generated data, we evaluated the expression of skin-enriched genes emerged from a transcriptomic and proteomic profiling study performed in 2015 [14]. As shown in Fig. 3, for three representative transcripts (*COL17A1*: Collagen Type XVII Alpha 1 Chain; *DMKN* Dermokine and *KRT10*: Keratin 10), a higher expression was detected in all the four skin components compared to non-skin tissue (high quality RNA collected from whole blood of 20 subjects and processed with the same sequencing protocol used for processing the skin components) (p < 0.001). A similar trend was obtained for other analyzed skin-related genes (data not shown).

## Discussion

The present study is aimed to implementing and optimizing a protocol for the extraction of RNA from different components of skin biopsy to generate transcriptomics data. The skin is innervated by sensory nerve fibers originating from the primary sensory neurons of dorsal root ganglia, and their degeneration inversely correlates with the severity of the peripheral neuropathy and the risk of developing neuropathic pain [15, 16]. Our hypothesis is that a modification at the transcriptome level could occur in the neighbouring environment of skin nerve fibers involved in the transduction of nociception, including Schwann cells, endothelial, interstitial, and inflammatory cells, as a consequence of pathology. According to this hypothesis, we can speculate that transcriptomic data generated in this study using this protocol will allow identifying target genes and expression markers of the cellular components involved in the disease (e.g. neuropeptides acting on neuronal and non-neuronal target cells). As a proof-of-concept, in our data we compared the expression levels of skin-specific genes (*COL17A1*, *DMKN* and *KRT10*) among skin compartments and whole blood as non-skin tissue, demonstrating a significant difference. To further explore our hypothesis, we considered the ELF in the protocol and in the analysis, because it is the component of greatest interest for detecting a transcriptional signature of peripheral neuropathy since it is enriched of intraepidermal nerve fibers selectively degenerated in the disease and which density is routinely counted in skin biopsy to diagnose the disease.

**Fig. 4:**
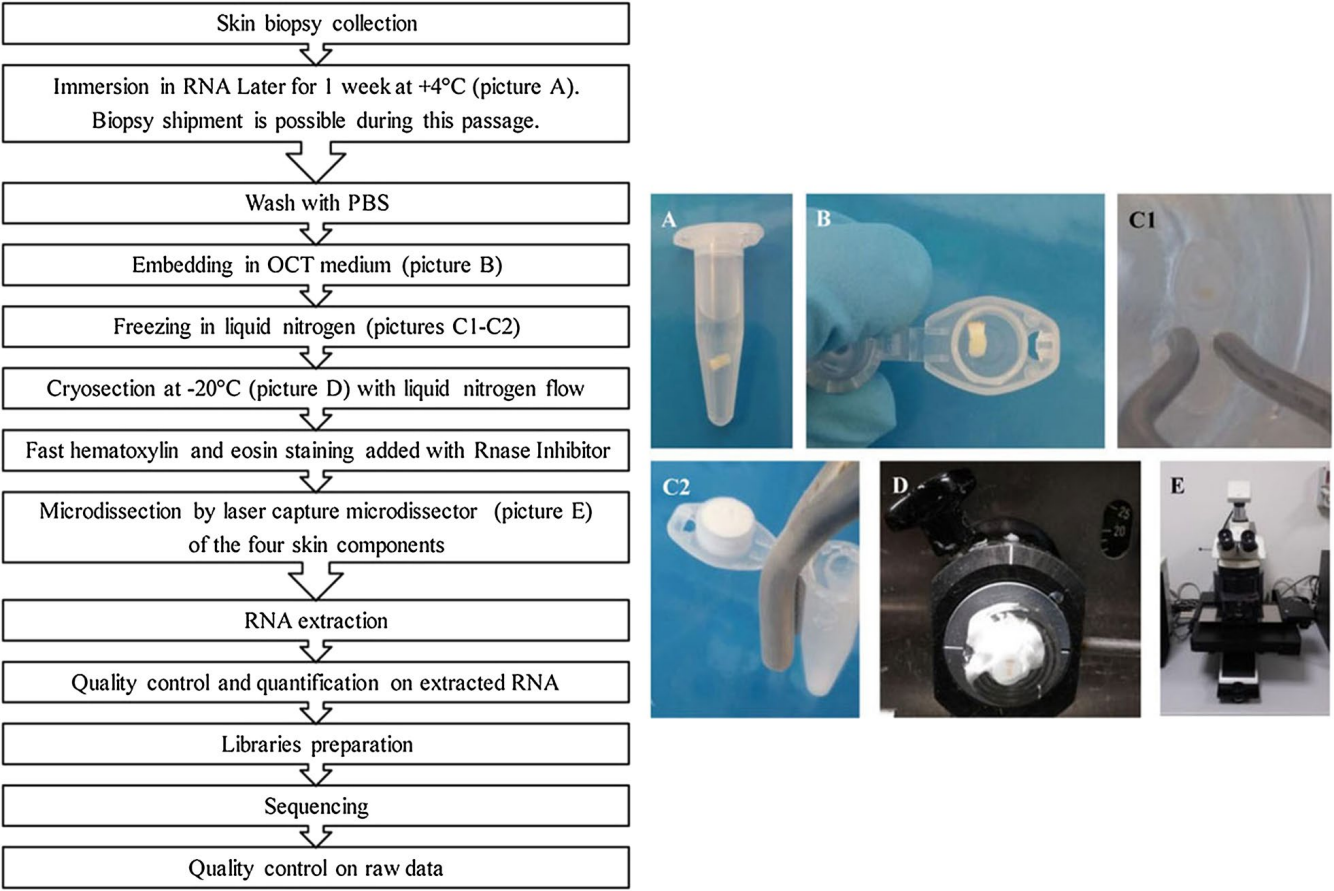
Workflow of the steps of the protocol. The flowchart summarizes the steps of the protocol from the skin biopsy collection to the quality control check on raw data generated from sequencing

Our protocol was optimized to reduce the impact of several events that affect the quality and the yield of RNA such as the activation of nucleases induced by the excision of the tissue [12], the presence of RNase and other deleterious components in aqueous environment when specimen is defrosted and in the aqueous reagents for sample preparation [7, 12], temperature changes from 4 °C to room temperature steps [9] and to higher temperatures generated by the energy of the laser during LMD. In Fig. 4 we reported a flowchart summarizing the steps of the protocol from skin biopsy collection to quality control steps on RNA sequencing raw data.

To preserve the RNA integrity, different solutions were evaluated. Brown et al. [10] suggested the use of high-salt buffer solutions during the immunostaining of rat brain sections to obtain high yield and quality RNA from cells isolated by microdissection. The authors concluded that an overnight incubation in 2M NaCl or RNALater^®^ Solution resulted in less degradation, although they preferred to avoid the use of the RNALater^®^ Solution because it affects the immunolabeling downstream process. However, in our protocol the use of RNALater^®^ Solution did not affect samples when they were examined for standard histological criteria [17], therefore we immersed the fresh biopsy in the solution to minimize the RNA degradation and the alteration of the expression profile immediately after the biopsy collection. In addition, this step performed in the first phases of the protocol facilitates the collection of specimens from distant laboratories in the context of multicenter studies allowing the shipment of specimens immersed in RNALater^®^ Solution under refrigerated condition. Our protocol was set up on 7 days of RNALater^®^ Solution incubation; however, few hours up to 1 month at 4 °C did not impact the RNA preservation.

LMD allows isolating homogenous tissues from which nucleic acids and proteins can be extracted for downstream analyses [6]. A good sample preparation starting with its inclusion in embedding medium, the cut of thin sections, and the staining to visualize tissue components are fundamental for the efficiency of dissection. Skin is the outermost organ of the mammalian with the main roles of functional barrier, temperature regulation, prevention of body water loss and protection of internal tissues and organs against external physical, chemical and biological insults [18–21]. It is a complex stratified tissue in which different cells organized in layers can be distinguished by expression markers and morphology [22] using immunolabeling and/or histological staining. The specific characteristics of the samples can be preserved choosing among different available embedding conditions. One of the most used tissue preservation method is the formalin fixation and paraffin embedding which allow the best morphological identification of tissue compartments [12]. In clinical practice and in pathological studies, formalin and paraffin fixations are commonly used, but they are not recommended for our aim because they chemically alter the nucleotides inducing nucleic acids fragmentation and lowering the quality of RNA [12]. For this reason, we opted for the cryopreservation of the tissue embedding the specimen with Killik cryostat in OCT medium. To overcome the difficulty of skin cryosection due to its tendency to thaw after freezing and to soften because of the high degree of fatty tissue, we facilitated the cut scrolling a little amount of liquid nitrogen over the sample positioned on the object holder of the cryostat every two cryosected sections to harden the biopsy and to decrease its temperature.

H&E staining was performed following the protocol published by Yee and colleagues [9] with some modifications, as reported in “Materials and methods”. As previously reported in different protocols of RNA extraction from pancreas, lymph node, breast and others tissues, the treatment with RNase Inhibitors minimized the degradation of RNA during immunohistochemistry and/or histological staining, during RNA extraction process and after RNA isolation [7, 10, 12]. For this reason, we added RNasin^®^ Plus RNase Inhibitor (Promega, Madison, WI, USA) in H&E solutions [10, 12], but not in final eluted RNA to avoid interference during libraries processing. Comparing with the original staining procedure, we performed an efficient coloration of the skin section in approximately 3 min instead of 5, diluting colored solution and adding inhibitors.

The hardness of the skin, mainly composed by keratinocytes and cornified layers, increases the risk of flaking of the outer epidermal layers during the laser capture microdissection. A specific balance between the cutting parameters of power, thickness, and velocity of the laser beam were adjusted to avoid the tissue break and to accelerate the cutting process limiting the RNA degradation due to the high temperature of laser.

Despite the introduction of several optimization steps to reduce RNA degradation, some samples did not fulfill Illumina recommendations regarding the RNA input for the RNA sequencing profiling (amount > 20 ng for samples with DV_200_ ≥ 70%, Additional file 1: Table S1). However, we proceed with library preparation, and in 80% of samples we were able to generate high quality libraries for sequencing, while for the remaining 20% we did not obtain enough cDNA to perform hybridization (Additional file 1: Table S1). We observed a positive correlation between the input material and the cDNA amount (Fig. 2a). The different yield of cDNA among samples impacted on the multiplexed modality of the hybridization since we were not able to hybridize 200 ng of each sample in all pool according to the manufacturer’s protocol. As expected, the yield of library was directly proportional to the amount of cDNA pooled (Fig. 2b). Despite the encountered limitations, the generated libraries fulfilled the requested quality and quantity parameters for sequencing, confirming that it is possible to generate high-quality libraries even starting with 17 ng of initial RNA.

The quality control steps of RNA sequencing data are critical to provide additional perspective into the quality of downstream results and to define the reliability and reproducibility of raw data [2]. As reported in Additional file 2: Figures S1A, B, the base sequence quality by cycle was higher than the common considered threshold of Q30 which is equivalent to the probability of an incorrect base call 1 in 1000 times. A typical shape of the Q score trend was observed with a smaller set of quality values observed at the first cycles reflecting the adjustment in the calibration of the sequencer while the drop of qualities at the end was a direct consequence of the final saturation and of the sequencing’s chemistry. The quality of base call was not influenced by tissue compartments (Additional file 2: Figures S1A) and by flow cells used (Additional file 2: Figures S1B), confirming the absence of batch effects caused by the nature of the samples and/or by the technique. Moreover, the uniformity of the samples of the same tissue compartment sequenced in different flow cells allowed to merge data and to perform downstream analyses without any impact on the quality of the entire experiment. We next performed a PCA analysis to confirm that samples of the same tissue clustered together more than different tissues from same individual (Additional file 4: Figure S3), and to identify potential outliers and batch effect. As a matter of fact, we identified two samples (S7d and S11g) that were deemed outliers and they were excluded from downstream analysis (Additional file 4: Figure S3).

The percentage of mapped reads was used as an additional indicator of the overall capture efficiency [23]. Although the percentage of mapped reads across samples were high and homogeneous, even though libraries were generated in limiting conditions of quality and quantity of the input material, a positive trend was observed between the degree of degradation and the capturing efficiency (Additional file 5: Figure S4B).

## Conclusions

In the present manuscript we describe an implemented and optimized protocol that allows obtaining reliable RNA sequencing data from fresh-frozen skin human biopsy by combining the LMD technology to NGS technology. Crucial steps of optimization of the protocol were an initial treatment of the specimens with RNALater^®^ preserving solution, the storage of the specimen at low temperature and using liquid nitrogen flow during cryosection, the addition of RNase inhibitors during H&E staining, and the time reduction of the staining procedure. The successful parameters of quality control and the overall homogeneity among the different samples, confirm that the protocol was effective in extracting RNA suitable for transcriptomic approach. Mostly, the implemented protocol allows generating expression profile starting with degraded RNA, and in particular in conditions in which the quality and quantity of extracted RNA was below suggested thresholds for the enrichment protocol. Downstream differential expression analysis will allow comparing the transcriptome in specific skin compartments of different subgroups of neuropathic patients (e.g. painful and painless) and healthy controls. Lastly, the implemented protocol can be used also in other diseases that require to collect biopsies from skin or other tissues, as well as in collaborative projects that require sampling from different centers, offering the opportunity to enlarge sample size for transcriptomics studies.

## Materials and methods

### Subject recruitment and Ethics Committee

Skin biopsies from 14 individuals (4 subjects affected by painful and 4 by painless idiopathic sensory neuropathy and 6 age- and sex-matched healthy controls) were collected at the IRCCS Foundation “Carlo Besta” Neurological Institute in Milan in agreement with the approval of the local Ethics Committee (Ethics Committee Number 03, 11/12/2013). Written consent was obtained from all recruited participants.

### Skin biopsy collection and preservation

Skin biopsies were taken using a disposable 3-mm punch 10 cm above the lateral malleolus under sterile condition after topic anesthesia with spray ice [24, 25]. Immediately after the sampling, the biopsy was submerged in 1.5 ml of RNALater^®^ Solution (Applied Biosystems, Foster City, CA, USA) and stored for 1 week at 4 °C to allow the permeation of the solution into cells resulting in RNA stabilization (Fig. 4) [26]. The following step was a double rinse of the biopsy in refrigerated DPBS 10× without Calcium and Magnesium (Sigma-Aldrich, St. Louis, MO, USA). The sample was then included in Killik cryostat embedding OCT medium (Bio Optica, Milan, Italy), snap-frozen and stored in liquid nitrogen for at least 1 week before proceeding to the next steps. RNase contamination was avoided by previously cleaning surfaces and instruments with RNaseZap^®^ Solution (Thermo Fisher Scientific, Waltham, MA, USA).

### Cryosection

The frozen samples were cryosected using the Leica cryostat (Leica Mikrosysteme Vertrieb, Wetzlar, Germany) set at − 20 °C. The blade was previously cleaned with RNaseZap^®^ Solution. Around 20–30 slices of 20 μm of thickness were transferred on Pen Membrane Glass Slides (Leica Mikrosysteme Vertrieb, Wetzlar, Germany) previously activated by exposure to UV light for 20 min. Every two cryosected slides a little amount of liquid nitrogen was scrolled over the sample positioned on the object holder to harden it. The tissue slices on the glass were stored at − 80 °C before proceeding with the histological coloration.

### Histological coloration

In order to recognize the skin layers of the biopsy, a fast hematoxylin and eosin staining was performed on the slices with slight modifications of the protocol published by Yee and colleagues [9]. Staining was performed after removing the glass slides from − 80 °C and leaving them at room temperature for few minutes to dry. Tissues were dehydrated in 70% ethanol (prepared in DEPC water) for 20 s. For the hematoxylin staining, 500 μl of diluted 1:2 hematoxylin solution in ethanol added with 1 U/μl RNasin^®^ Plus RNase Inhibitor (Promega, Madison, WI, USA) shortly before the use [10, 12], was used for 10 s. After a 10 s wash in DEPC water and an additional 10 s wash in 70% ethanol, the slices were stained for 2 s with 250 μl of diluted 1:2 eosin solution in ethanol containing 1 U/μl RNasin^®^ Plus RNase Inhibitor (Promega, Madison, WI, USA) shortly before the use. Instead of staining the glass slides by immersion, they were horizontally positioned on the bench and drops of colored solutions were added over. Subsequently, after a 10 s wash in DEPC water and a 10 s wash in 70% ethanol, the tissues on glass slides were dried at room temperature for few minutes and then stored at − 80 °C before proceeding with the laser microdissection.

### Laser microdissection

The colored slides were removed from − 80 °C and left few minutes at room temperature to dry before being placed on a Leica Microdissection system LMD6000 (Leica Mikrosysteme Vertrieb, Wetzlar, Germany). To minimize the damage of the laser on the tissue, the focus and the energy of the laser were modified for each slide. Cut sections fell by gravity in the tube and were stored at − 80 °C before proceeding with the RNA extraction.

### Type and dimension of microdissected tissues

From the 20–30 colored tissue slices of each subject, four different microdissected skin components were collected: (a) the enriched layer of fibers extending from the epidermis to 200–300 μm deep into the biopsy; (b) the glands; (c) the dermis, and (d) the whole section. No relative percentage of cell types was measured, and no specific recognition was performed to determine the differences between exocrine and endocrine glands. Moreover, fat cells were not removed from the glandular tissue during the microdissection.

### RNA extraction and DNase treatment

Extraction of total RNA was performed using the PicoPure^®^ RNA Isolation Kit (Thermo Fisher Scientific, Waltham, MA, USA). As first step, the addition of extraction buffer was performed with 100 μl for each tissue except for glands, for which 50 μl were added to avoid an excessive dilution of RNA due to the small amount of microdissected material. An on-column DNase digestion was performed treating the RNA bound to the column for 20 min with 5 μl of DNase I diluted in 35 μl of RDD buffer (RNase-Free DNase Set, Qiagen, Valencia, CA) to remove genomic DNA. 20 μl of RNase Nuclease free water for all tissues, except for glands for which 15 μl was used, were pipetted directly onto the membrane of the purification column to elute the RNA into an RNase free tube. RNA was then stored at − 80 °C.

### Quality control and concentration measurements of the extracted RNA

RNA quality was estimated by microfluidic capillary electrophoresis by running 1 μl of each sample on an Agilent 2100 Bioanalyzer Instrument (Agilent Technologies, California, United States) using the Agilent RNA 6000 Pico Kit. The RNA degradation was evaluated measuring the percentage of RNA fragments longer than 200 nucleotides and the RNA Integrity Number. The concentration of RNA was measured by fluorometric quantitation using the Qubit^®^ RNA HS Assay kit on Qubit^®^ 2.0 Fluorometer (Thermo Fisher Scientific, Waltham, MA, USA).

### Libraries preparation and sequencing

RNA was processed for library preparation using the TruSeq^®^ RNA Access Library Preparation Kit (Illumina, San Diego, CA, USA) that allows generating libraries starting from degraded and low yield RNA. Briefly, the first and second cDNA strands were synthetized from input RNA in order to be adaptor-tagged, labeled and amplified. CDNA from each sample was then pooled. Pooling strategy was decided considering tissues and subjects uniformity. Each pool contained two to four samples of the same tissue and included both patients and controls. Pooled samples were enriched by a double step of probes hybridization. The enriched targets were captured by streptavidin labelled beads, cleaned up and amplified to obtain the final multiplexed libraries. Libraries quality was checked for fragments distribution using Agilent DNA High Sensitivity Kit on an Agilent 2100 Bioanalyzer Instrument (Agilent Technologies, Santa Clara, CA, USA) and quantified by Qubit^®^ DNA HS Assay kit on a Qubit^®^ 2.0 Fluorometer (Thermo Fisher Scientific, Waltham, MA, USA). The libraries were then sequenced on an Illumina HiSeq^®^ 2500 platform (Illumina^®^) with a rapid paired-end sequencing (2 × 101 bp). The protocol for libraries generation was executed step by step except for the adjustment of cDNA amount used for hybridization (details shown in the results section).

### Quality control analysis of RNA sequencing

The quality control check on raw sequenced data was assessed with FastQC (version 0.11.3) in a non-interactive mode [27]. The obtained numeric values were aggregated to generate a unique plot of base sequence quality averaging the score and the standard deviation of sequenced bases stratified for tissue compartments and flow cells. Since some of the samples had an amount of reads > 45 million compared to the median value, we performed a down sampling step, reducing their amount to 35 million using the seqtk software (version 0.2.2). Reads were then trimmed and short reads were removed using Skewer 0.2.2 tool [28]. We carried out Principal Component Analysis to assess overall grouping of data according to tissue type and to detect potential outliers. PCA was applied to the matrix of down-sampled log10-transformed raw counts. Pair-end reads were aligned using STAR software (version 2.5.2b) [29] against the GRCh37 reference genome. Quantification of genes and isoforms was performed with RSEM software version 1.3.3 [30] using the ENSEMBL GRCh37.87 annotation GTF file.

### Statistical analyses

Statistical data and graphs were performed in GraphPad Prism Software, version 5.04. Mean values and standard deviations are presented for different measures. Correlation analysis was carried out with the Spearman’s r coefficient calculation. All the other data were analyzed statistically by the Mann–Whitney nonparametric test. Differences were considered statistically significant when the p value was ≤ 0.05.

## Additional files

**Additional file 1: Table S1.** Summary of characteristics of subjects and skin components. Quality parameters on extracted RNA (RIN and DV_200_), RNA input amount used for library preparation, RNA concentration of pre-pooled samples, amount of hybridized cDNA, final concentration of libraries and million of reads sequenced for sample are shown. Subjects are indicated by the sample ID. Samples from number 1 to 8 were patients and from number 9 to 14 were healthy controls. Samples pooled together are indicated by the same capital letter (from A to O) in the pool row. The samples that did not reach the requested amount for hybridization were excluded from the experiment (marked as “X”).

**Additional file 2: Figure S1.** Base sequence quality score. The two graphs show the Phred quality score (Q score) on y axis across all sequenced based on x axis at each position in the FASTQ file for the different skin layers, flow cells (FC) and reads (1 and 2). All the values were reported as mean ± standard deviation. A) Q score distribution across different tissues (ELF: enriched layer of fibers, G: glands, D: dermis and WS: whole section); B) Q score distribution across the different FC used for sequencing.

**Additional file 3: Figure S2.** Density plot. Density plot of log10-transformed reads counts of protein coding genes is reported. The global trend shows a distribution close to a Gaussian distribution and a similarity across all samples. ELF: enriched layer of fibers, G: glands, D: dermis and WS: whole section.

**Additional file 4: Figure S3.** Principal Component Analysis (PCA). PCA was performed on log10-transformed down-sampled reads counts. The first 3 principal components (PCs), explaining the 43% of the variance, are shown. For each sample, the color and the label indicate the tissue (elf: enriched layer of fibers in pink; g: glands in blue; d: dermis in green and ws: whole section in yellow), while the number is related to the subject ID.

**Additional file 5: Figure S4.** Percentage of uniquely mapped reads. A) Uniquely mapped reads reported in percentage for each tissue (ELF enriched layer of fibers; G glands; D dermis and WS whole section). Scatter dot plot shows the mean ± standard deviation and each dot represents the value of a single sample. Numeric values are reported for each tissue as mean ± standard deviation. B) Relationship between the uniquely mapped reads and RNA degradation expressed as DV_200_ (p: 0.013, beta: 0.11 and r^2^: 0.15). Each dot represents one sample.

## Abbreviations

LMD: laser capture microdissection
DV_200_: fragments longer than 200 nucleotides
RIN: RNA Integrity Number
NGS: next generation sequencing
H&E: hematoxylin and eosin
ELF: enriched layer of fibers
G: glands
D: dermis
WS: whole section
SD: standard deviation
Q score: quality score
PCA: Principal Component Analysis
PC: principal component
