# Supplementary material for: Laser capture microdissection for transcriptomic profiles in human skin biopsies
Source: BMC Mol Biol. 2018 Jun 19;19:7. doi: 10.1186/s12867-018-0108-5 (PMC6009967; doi:10.1186/s12867-018-0108-5)
Supplement: Supplementary file 1 — Additional file 1: Table S1. Summary of characteristics of subjects and skin components. Quality parameters on extracted RNA (RIN and DV200), RNA input amount used for library preparation, RNA concentration of pre-pooled samples, amount of hybridized cDNA, final concentration of libraries and million of reads sequenced for sample are shown. Subjects are indicated by the sample ID. Samples from number 1 to 8 were patients and from number 9 to 14 were healthy controls. Samples pooled together are indicated by the same capital letter (from A to O) in the pool row. The samples that did not reach the requested amount for hybridization were excluded from the experiment (marked as “X”). [file 12867_2018_108_MOESM1_ESM.docx]

**ADDITIONAL FILE**

**Additional Table S1. Summary of characteristics of subjects and skin components.**

| **Enriched layer of fibers** | | | | | | | | | | | | | | |
| --- | --- | --- | --- | --- | --- | --- | --- | --- | --- | --- | --- | --- | --- | --- |
| *sample ID* | S1 | S2 | S3 | S4 | S5 | S6 | S7 | S8 | S9 | S10 | S11 | S12 | S13 | S14 |
| *RIN* | 1.9 | 2.3 | 2.1 | 1.8 | 2.2 | 2.2 | 2.2 | 2.6 | 2.2 | 2.2 | 2.3 | 2.7 | 1.1 | 2.7 |
| *DV_200_* | 83 | 90 | 94 | 92 | 84 | 82 | 84 | 90 | 91 | 92 | 80 | 97 | 75 | 94 |
| *RNA input (ng)* | 17 | 20 | 20 | 20 | 20 | 20 | 17 | 20 | 20 | 20 | 17 | 20 | 17 | 20 |
| *concentration pre-hybridization (ng/µl)* | 5.6 | 4.7 | 55 | 7.4 | 49.4 | 55 | 49.4 | 48.7 | 56 | 0.1 | 49.5 | 47.8 | 0.3 | 54 |
| *hybridized amount (ng)* | 60 | 60 | 200 | 60 | 200 | 200 | 200 | 200 | 200 | X | 200 | 200 | X | 200 |
| *Pool* | A | A | B | A | C | B | D | D | D |  | B | C |  | C |
| *library concentration (ng/µl)* | 1.5 | 1.5 | 9.2 | 1.5 | 6.5 | 9.2 | 5.2 | 5.2 | 5.2 |  | 9.2 | 6.5 |  | 6.5 |
| *sequenced reads (million)* | 33.6 | 45.1 | 58.7 | 17.8 | 26.9 | 27.8 | 40.1 | 49.1 | 46.2 |  | 41.9 | 51.7 |  | 56.3 |

| **Glands** | | | | | | | | | | | | | | |
| --- | --- | --- | --- | --- | --- | --- | --- | --- | --- | --- | --- | --- | --- | --- |
| *sample ID* | S1 | S2 | S3 | S4 | S5 | S6 | S7 | S8 | S9 | S10 | S11 | S12 | S13 | S14 |
| *RIN* |  | 2.4 |  |  | 2.9 | 1.6 | 1 | 1 | 1 | 1 | 1 | 1 |  |  |
| *DV_200_* |  | 76 |  |  | 82 | 83 | 88 | 80 | 87 | 68 | 79 | 86 |  |  |
| *RNA input (ng)* |  | 17 |  |  | 19 | 17 | 17 | 17 | 17 | 17 | 17 | 17 |  |  |
| *concentration pre-hybridization (ng/µl)* |  | 10.7 |  |  | 52 | 33.6 | 12.8 | 4.9 | 20.4 | 10.2 | 3 | 12.5 |  |  |
| *hybridized amount (ng)* |  | 60 |  |  | 60 | 60 | 60 | 35 | 60 | 60 | 35 | 60 |  |  |
| *Pool* |  | E |  |  | F | F | E | G | F | F | G | E |  |  |
| *library concentration (ng/µl)* |  | 2.2 |  |  | 5.7 | 5.7 | 2.2 | 0.4 | 5.7 | 5.7 | 0.4 | 2.2 |  |  |
| *sequenced reads (million)* |  | 40.6 |  |  | 89.8 | 34.8 | 50.4 | 26.1 | 31.4 | 23.6 | 29.4 | 43.9 |  |  |

| **Dermis** | | | | | | | | | | | | | | |
| --- | --- | --- | --- | --- | --- | --- | --- | --- | --- | --- | --- | --- | --- | --- |
| *sample ID* | S1 | S2 | S3 | S4 | S5 | S6 | S7 | S8 | S9 | S10 | S11 | S12 | S13 | S14 |
| *RIN* | 1.3 | 1 | 1.1 | 1 | 1 | 2.2 | 1 | 1 | 1 | 1.4 | 1 | 1 | 1 | 1 |
| *DV_200_* | 78 | 61 | 89 | 87 | 82 | 84 | 72 | 82 | 67 | 89 | 67 | 89 | 77 | 83 |
| *RNA input (ng)* | 17 | 17 | 17 | 20 | 20 | 17 | 17 | 20 | 20 | 17 | 17 | 20 | 20 | 20 |
| *concentration pre-hybridization (ng/µl)* | 4.8 | 2.2 | 2.3 | 0.1 | 0 | 0.2 | 1.1 | 0.1 | 0.1 | 7.1 | 0.1 | 4.9 | 0.1 | 0.5 |
| *hybridized amount (ng)* | 60 | 27 | 27 | X | X | 60 | 27 | X | X | 60 | X | 60 | X | X |
| *Pool* | H | I | I |  |  | H | I |  |  | H |  | H |  |  |
| *library concentration (ng/µl)* | 1.8 | 0.7 | 0.7 |  |  | 1.8 | 0.7 |  |  | 1.8 |  | 1.8 |  |  |
| *sequenced reads (million)* | 46.1 | 22.3 | 37.5 |  |  | 52 | 13.2 |  |  | 38.1 |  | 50.7 |  |  |

| **Whole section** | | | | | | | | | | | | | | |
| --- | --- | --- | --- | --- | --- | --- | --- | --- | --- | --- | --- | --- | --- | --- |
| *sample ID* | S1 | S2 | S3 | S4 | S5 | S6 | S7 | S8 | S9 | S10 | S11 | S12 | S13 | S14 |
| *RIN* | 1.9 | 5.4 | 1 | 1.7 | 2.5 | 2.4 | 2.3 | 4.2 | 2.4 | 2.5 | 2 | 3.2 | 1 | 2.7 |
| *DV_200_* | 78 | 93 | 93 | 91 | 85 | 84 | 86 | 85 | 86 | 90 | 81 | 97 | 81 | 94 |
| *RNA input (ng)* | 17 | 19 | 17 | 17 | 20 | 20 | 20 | 17 | 20 | 20 | 20 | 20 | 17 | 19 |
| *concentration pre-hybridization (ng/µl)* | 7 | 28.3 | 16.5 | 2.8 | 55 | 42.9 | 53 | 10.2 | 58 | 52 | 28.2 | 68.6 | 0.5 | 62.8 |
| *hybridized amount (ng)* | 87.5 | 200 | 200 | 27 | 200 | 200 | 200 | 87.5 | 200 | 200 | 200 | 200 | X | 200 |
| *Pool* | L | M | N | I | N | M | O | L | M | N | N | O |  | M |
| *library concentration (ng/µl)* | 1.8 | 4.7 | 3.5 | 0.7 | 3.5 | 4.7 | 6.1 | 1.8 | 4.7 | 3.5 | 3.5 | 6.3 |  | 4.7 |
| *sequenced reads (million)* | 62.8 | 35.0 | 28.6 | 31.0 | 34.2 | 17.8 | 56.3 | 81.2 | 31.0 | 31.9 | 21.6 | 88.5 |  | 36.1 |

**Quality parameters on extracted RNA (RIN and DV_200_), RNA input amount used for library preparation, RNA concentration of pre-pooled samples, amount of hybridized cDNA, final concentration of libraries and million of reads sequenced for sample are shown. Subjects are indicated by the sample ID. Samples from number 1 to 8 were patients and from number 9 to 14 were healthy controls. Samples pooled together are indicated by the same capital letter (from A to O) in the pool row. The samples that did not reach the requested amount for hybridization were excluded from the experiment (marked as “X”).**
