# Supplementary figures and images for: Laser capture microdissection for transcriptomic profiles in human skin biopsies
Source: BMC Mol Biol. 2018 Jun 19;19:7. doi: 10.1186/s12867-018-0108-5 (PMC6009967; doi:10.1186/s12867-018-0108-5)

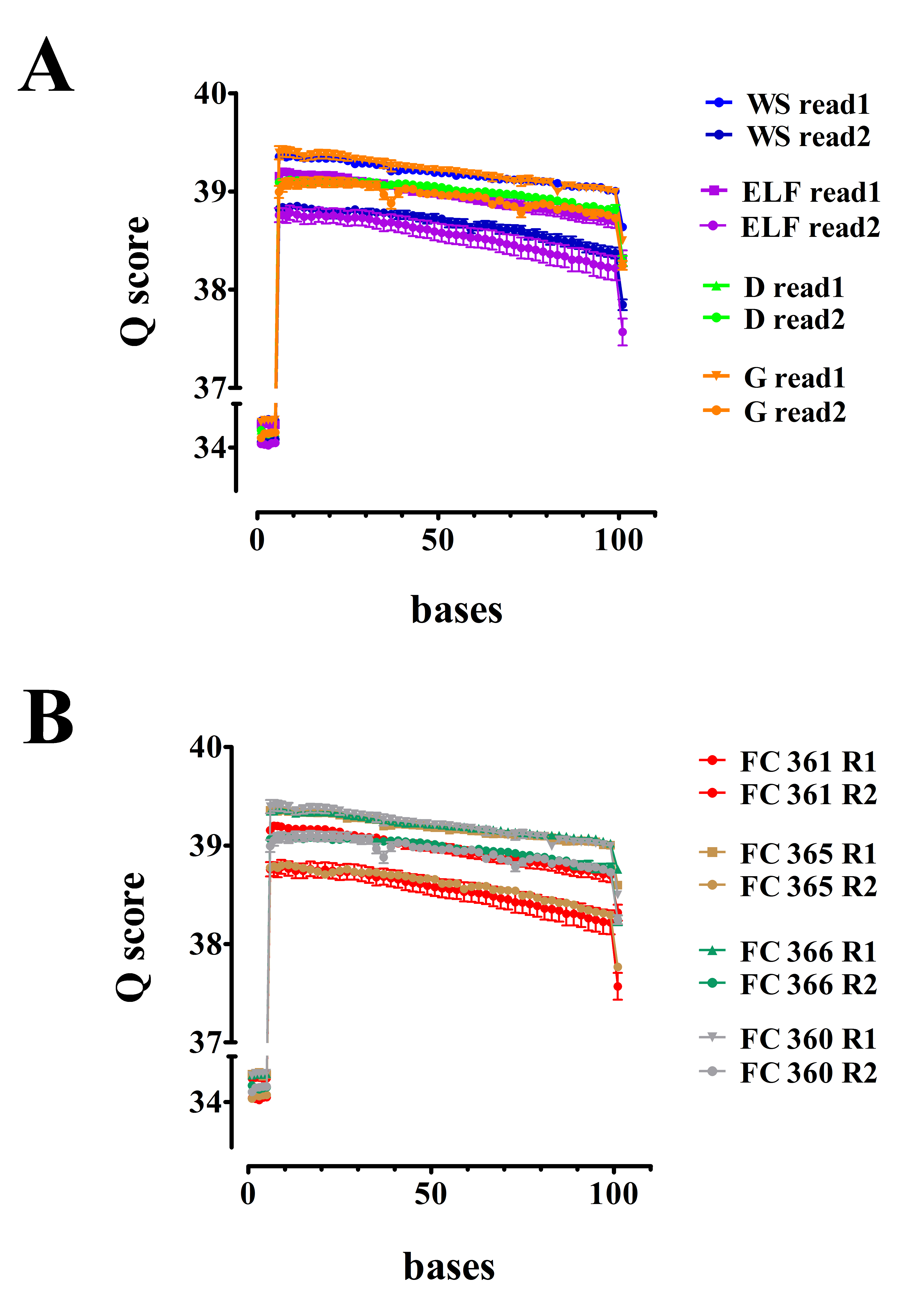

Supplement: Supplementary file 2 — Additional file 2: Figure S1. Base sequence quality score. The two graphs show the Phred quality score (Q score) on y axis across all sequenced based on x axis at each position in the FASTQ file for the different skin layers, flow cells (FC) and reads (1 and 2). All the values were reported as mean ± standard deviation. A) Q score distribution across different tissues (ELF: enriched layer of fibers, G: glands, D: dermis and WS: whole section); B) Q score distribution across the different FC used for sequencing. [file 12867_2018_108_MOESM2_ESM.jpg]

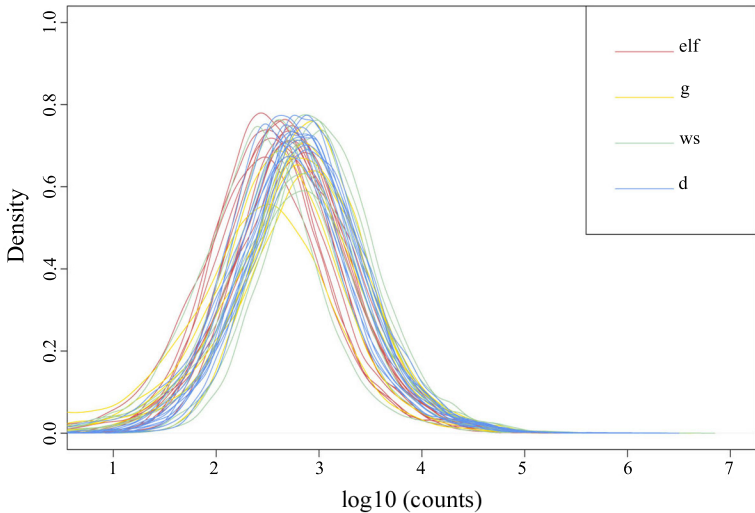

Supplement: Supplementary file 3 — Additional file 3: Figure S2. Density plot. Density plot of log10-transformed reads counts of protein coding genes is reported. The global trend shows a distribution close to a Gaussian distribution and a similarity across all samples. ELF: enriched layer of fibers, G: glands, D: dermis and WS: whole section. [file 12867_2018_108_MOESM3_ESM.pdf]

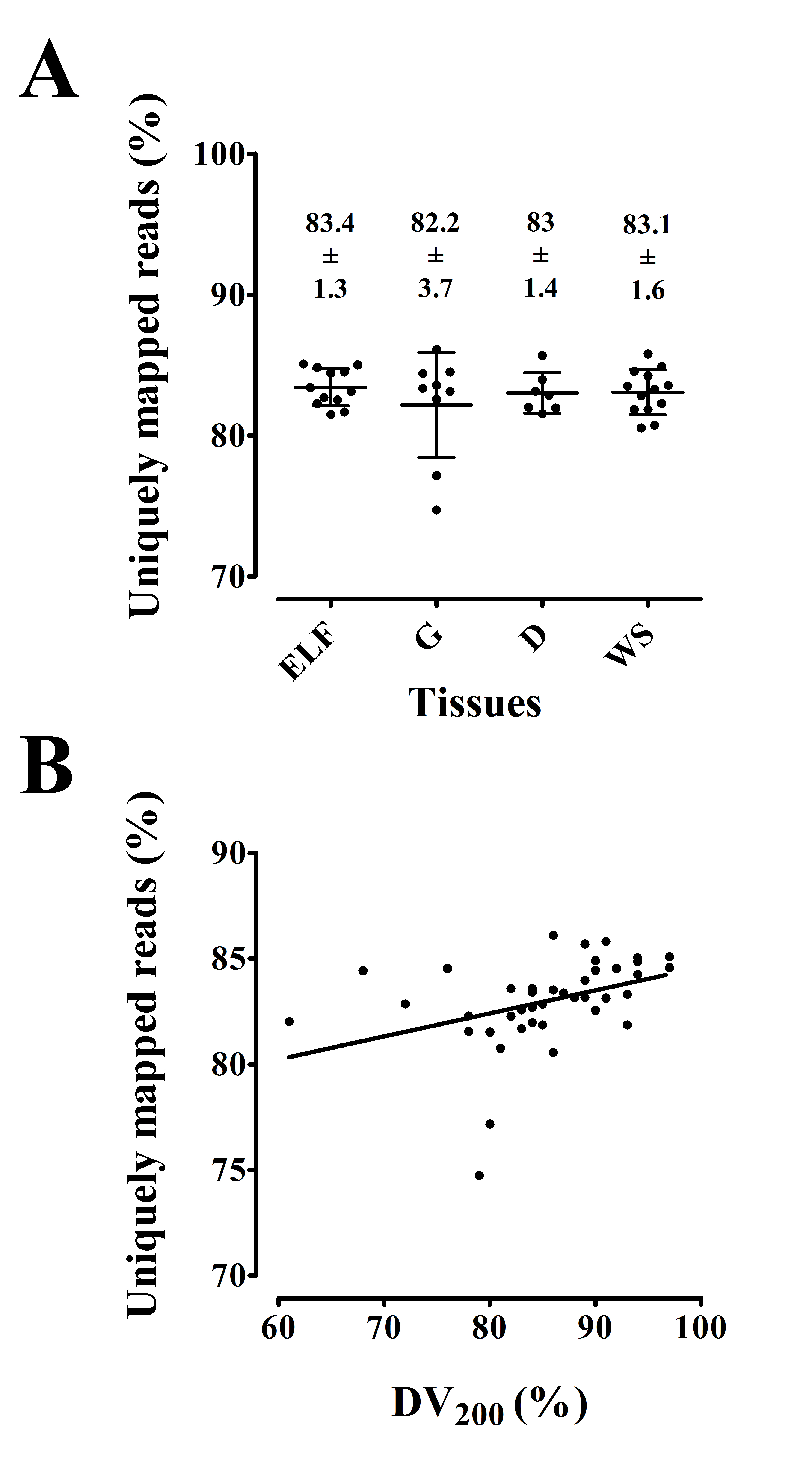

Supplement: Supplementary file 5 — Additional file 5: Figure S4. Percentage of uniquely mapped reads. A) Uniquely mapped reads reported in percentage for each tissue (ELF enriched layer of fibers; G glands; D dermis and WS whole section). Scatter dot plot shows the mean ± standard deviation and each dot represents the value of a single sample. Numeric values are reported for each tissue as mean ± standard deviation. B) Relationship between the uniquely mapped reads and RNA degradation expressed as DV200 (p: 0.013, beta: 0.11 and r2: 0.15). Each dot represents one sample. [file 12867_2018_108_MOESM5_ESM.jpg]
