# Supplementary material for: Laser capture microdissection for transcriptomic profiles in human skin biopsies
Source: BMC Mol Biol. 2018 Jun 19;19:7. doi: 10.1186/s12867-018-0108-5 (PMC6009967; doi:10.1186/s12867-018-0108-5)
Supplement: Supplementary file 4 — Additional file 4: Figure S3. Principal Component Analysis (PCA). PCA was performed on log10-transformed down-sampled reads counts. The first 3 principal components (PCs), explaining the 43% of the variance, are shown. For each sample, the color and the label indicate the tissue (elf: enriched layer of fibers in pink; g: glands in blue; d: dermis in green and ws: whole section in yellow), while the number is related to the subject ID. [file 12867_2018_108_MOESM4_ESM.pdf]

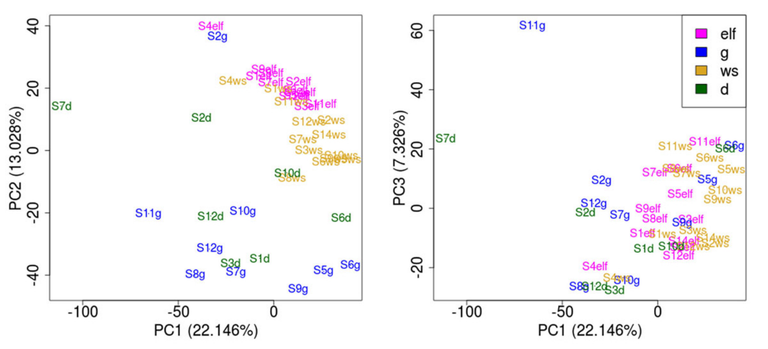
PC1 (22.146%)
PC2 (13.028%)
PC3 (7.326%)
elf
g
ws
d
S4elf
S2g
S9elf
S7elf
S2elf
S4ws
S7d
S2d
S11ws
S3elf
S12ws
S2ws
S14ws
S7ws
S3ws
S10ws
S6ws
S10d
S8ws
S11g
S12d
S10g
S6d
S12g
S3d
S1d
S8g
S7g
S5g
S6g
S9g
S11elf
S5ws
S5elf
S9ws
S8elf
S1elf
S12elf
S4ws
S10d
-100
-50
0
-40
-20
20
40
60
